# Ozone Risk Assessment and Mapping in the Alps Based on Data from Passive Samplers

**DOI:** 10.1100/tsw.2002.128

**Published:** 2002-04-17

**Authors:** Cristina Mazzali, Elisabetta Angelino, Giacomo Gerosa, Antonio Ballarin-Denti

**Affiliations:** Lombardy Foundation for the Environment, Piazza Diaz 7, 20121 Milan, Italy; A.R.P.A. Lombardia, via Juvara 22, 20129 Milan, Italy; University of Milan, Department of Plant Production, via Celoria 2, 20133 Milan, Italy; Catholic University of Brescia, Department of Mathematics and Physics, via Musei 41, 25121 Brescia, Italy

**Keywords:** ozone, risk assessment, Alps, forests, passive samplers, exposure indices, AOT40, geostatistics

## Abstract

Passive samplers (diffusion tubes with organic reagent, produced by Passam of Switzerland) were used in a sampling campaign for the detection of weekly mean ozone concentrations in 15 sites over a domain of 80 x 40 km on the southern side of the European Alps from May to August 1998. The area is characterized by vast natural terrain of complex topography, with conifer and broadleaf forests. It is difficult to access and monitor air quality there with continuous analysers. By applying geostatistical techniques (ordinary kriging), and correcting the interpolated ozone concentrations according to the altitude of each single grid cell (2 x 2 km), maps of weekly ozone concentrations were produced. The weekly ozone data were used to assess daily and hourly data by means of an iterative procedure based on a functional dependence of ozone concentrations both on altitude and on the time of day. This allowed the estimation of values with an exposure index such as AOT40 (accumulated exposure over the threshold of 40 ppb) in all 800 cells of the domain. This also allowed the mapping of risk assessment related to the effects of ozone on the regional forest vegetation. Results obtained show values that exceed the exposure standards adopted in the Kuopio protocol (1996). Excess exposure values also match values calculated over a wider territorial domain by using hourly data on ozone concentration derived from continuous automatic analysers.

## INTRODUCTION

The growing stress of high ozone levels in alpine forest ecosystems faces us with the need to perform risk assessment for specific receptors to allow for the protection of the ecological, economic, and aesthetic values of vegetation. During the assessment of dose-response relationship of vegetation to ozone exposure, investigators concentrated on the use of indices to quantify the exposure. Several studies have dealt with both definition and assessment of these indices, taking into account the most harmful pollution levels for vegetation[1,2]. The most frequently used exposure indices attribute different weights to high and low ozone concentrations; these indices include $SUM_x$ (sum of the hourly concentrations beyond the $x$ threshold), $AOT_x$ (sum of the differences between hourly concentrations and the $x$ threshold), $W_y$ (sum of the hourly concentrations weighted by means of sigma functions indicated by the value of $y$), and $AL_y$ (sum of the hourly concentrations weighted by means of allometric functions indicated by the value of $y$)[3].

According to some indices, literature supplies potential damage thresholds (critical levels) related to the action of ozone on forest vegetation.

In the European context, the most widely used index is the AOT40 (accumulated exposure over the threshold of 40 ppb). For forest vegetation, it is calculated as the sum of differences between hourly concentrations and the 40 ppb threshold. This was determined using data obtained by continuous measurements from April to September during light hours (global solar radiation over 50 $W/m^2$)[4]. The period from April to September was chosen to cover both the vegetative growth season and the period of most intense gaseous exchanges between atmosphere and plants. The level of potential damage for forests associated with AOT40 index (ozone critical level for forests) has been set to 10,000 ppbh[5]. We decided to use AOT40 due to its wide use in European policy making and guidelines for air quality standards.

According to the AOT40 definition, in order to calculate AOT40, all hourly data sampled from April to September must be available. Researchers should therefore rely on continuous analysers operating for long periods of time. Moreover, in order to achieve a proper assessment of the index over a wide territory, several measurement sites have to be available, so that the investigated domain can be properly covered.

Unfortunately, forest areas are mainly located in complex terrain and are not easy to cover entirely. These features sometimes make it impossible to set up measurement campaigns with continuous analysers. Moreover, in such terrain ozone concentration depends on altitude, which makes data processing necessary for the extension of point data information to the whole area under study (map processing being more difficult).

This difficulty in having long-term, continuous data sets available, along with the need for several measurement sites, can be solved by using alternate monitoring techniques. In this case, the use of passive samplers seems to answer to both requirements. In fact, this monitoring tool can both make a high number of measurement points available and guarantee the extension of the campaign in time, due to its easy installation, low maintenance, independence from an electric supply, and low costs. However, passive samplers provide an integrated value in time (on a weekly basis), while the calculation of the index involves the use of hourly data. The aim of this work has therefore been to provide an alternative methodology for the assessment of the AOT40 index on a terrain with complex topography on the basis of information supplied by passive samplers.

## EXPERIMENTAL METHODS AND COMPUTATIONAL PROCEDURES

### The Sampling Campaign

The area covered by the monitoring campaign is located in a mountain area south of the Alps in Italy and characterized by a wide valley oriented eastward with several side valleys (Valtellina).

This area was also chosen because of previous studies on tropospheric ozone and its effect on vegetation that were performed there in the last 10 years[6,7]. Furthermore, data collected over several years through monitoring campaigns by passive samplers were available[8]. Besides weekly concentration data provided by passive samplers, hourly concentration values were also available from continuous analysers placed in mobile labs[9] in the same sites and for long periods of time.

The monitoring campaign for this research was conducted by means of passive samplers from May to August 1998. The first aim was map production for weekly ozone concentrations covering the domain of 80 × 40 km mentioned above.

The number of measurement sites was set to 15. This was a compromise between the need to obtain interpolations at sufficient confidence for an area with such a complex morphology on one hand and the need to keep the project logistically and economically sustainable on the other.

Sites where passive samplers were placed were chosen according to specific criteria, such as the area's typical features, altitude (ranging from 300 to 2,000 m a.s.l.), and representative forest vegetation. Areas more than 2,000 m a.s.l. were excluded. The map in Fig. 1 shows location of all sampling sites. Passive samplers (from Passam, of Männedorf, Switzerland)[10] were exposed in each site for a week (2 weeks in a few cases). Table 1 shows concentration values sampled in the 15 selected sites over the whole campaign period.

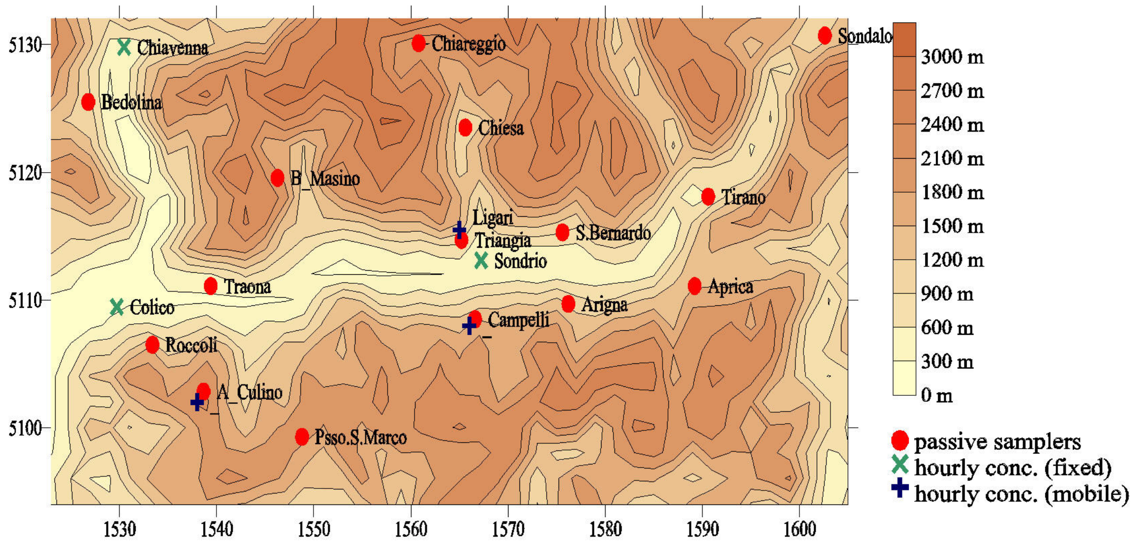

**FIGURE 1.** Site location map.

## TABLE 1
## Ozone Weekly Concentrations (ppb) Measured by Passive Samplers at the 15 Selected Sites

| | Traona | Tirano | Bedolina | Triangia | Arigna | Chiesa | Sondalo | S. Bernardo | Bagni Masino | Campelli | Alpe Culino | Roccoli | Aprica | Chiareggio | Psso. San Marco |
|---|---|---|---|---|---|---|---|---|---|---|---|---|---|---|---|
| 11/05-25/05 | 46,8 | 21,8 | 57,7 | 52,4 | 50,1 | 51,55 | 56,55 | 46,65 | 48,55 | 66,1 | 61,95 | 50,35 | 37 | 54,5 | 61,15 |
| 25/05-01/06 | 42,2 | 24,95 | 38 | 38,55 | 44,15 | 37,15 | 41,1 | 77,8 | 37,2 | 49,4 | 52,45 | 43,5 | 45 | 55,3 | 59,1 |
| 01/06-08/06 | 57,55 | 32 | 48,8 | 47,45 | 49,7 | 45,7 | 48,3 | 47,45 | 33,55 | 56,2 | 59,35 | 51,9 | 43,9 | 45,1 | 87,35 |
| 08/06-15/06 | 47,05 | 34,25 | 54,95 | 57,2 | 54,2 | 47,35 | 54,95 | 55,65 | 49,35 | 77,45 | 72,95 | 60,45 | 55,9 | 53 | 48,75 |
| 15/06-22/06 | 57,45 | 28,65 | 52,05 | 53,1 | 55,75 | 47,15 | 48,6 | 57,95 | 39,5 | 69,3 | 63,1 | 60,25 | 47,65 | 42,95 | 49,7 |
| 22/06-29/06 | 66,75 | 35,7 | 56,65 | 57,15 | 47,85 | 57,05 | 58,3 | 59,4 | 49,7 | 61,95 | 72,4 | 58,5 | 59,15 | 50,9 | 88,95 |
| 29/06-06/07 | 35,4 | 29,2 | 55 | 42,3 | 49,2 | 45,9 | 52,7 | 50,35 | 24,4 | 59,45 | 56,35 | 46,05 | 45,95 | 53,25 | 34,85 |
| 06/07-13/07 | 48,4 | 29,5 | 57,05 | 51,1 | 53,3 | 48,3 | 55,15 | 56,9 | 40,05 | 57,4 | 62,3 | 47,05 | 48,35 | 60,95 | 59 |
| 13/07-20/07 | 54,1 | 26,5 | 59,3 | 53,85 | 45,75 | 48,85 | 54,6 | 52,25 | 40,3 | 72 | 62,65 | 53,9 | 44,8 | 39,2 | 73,6 |
| 20/07-27/07 | 55,65 | 34,45 | 62,8 | 58,7 | 55,95 | 49,9 | 61,95 | 60,2 | 48,85 | 77,8 | 77,7 | 56,95 | 46,8 | 54,8 | 73,7 |
| 27/07-03/08 | 44,85 | 32,5 | 58,05 | 48,1 | 48,6 | 37,85 | 51,25 | 50,7 | 36,45 | 49,4 | 67,65 | 53 | 46,1 | 35,15 | 70,15 |
| 03/08-17/08 | 29,8 | 7,1 | 39,8 | 36,3 | 24 | 31,45 | 31,45 | 40,45 | 29,95 | 48,4 | 51,35 | 22,25 | 35,65 | 39,15 | 43,3 |
| 17/08-31/08 | 32,5 | 15,7 | 40,15 | 43,2 | 49,75 | 30 | | 40,3 | 32,7 | 47,6 | 52,65 | 35,6 | | 35 | 35,6 |

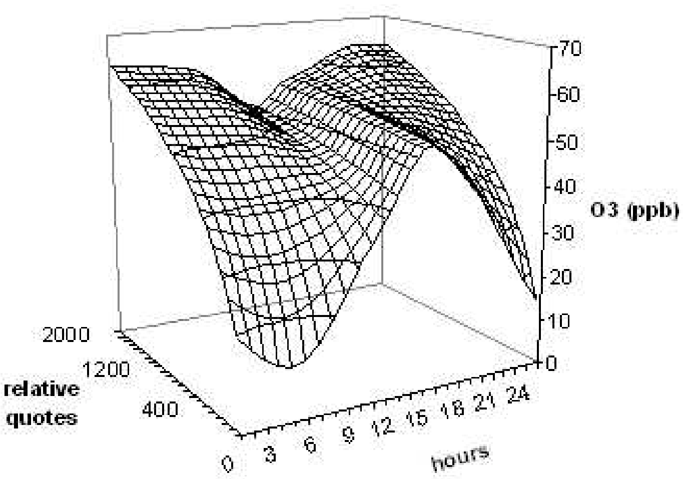

**FIGURE 2.** Ozone concentration dependence on altitude and daytime (Loibl function).

Besides the 15 sites where data were collected by passive samplers, weekly mean values were considered from two continuous monitoring stations (specifically, Colico and Chiavenna; see Fig. 2). We decided to consider these data in order to increase both the amount of information available and the precision of the interpolation.

## Ozone Maps

In order to calculate ozone concentration at all of the sites in the studied domain and to produce maps, geostatistical interpolation techniques have been applied based on the assumption that an interrelationship exists between the sampled ozone levels at different parts of the investigated terrain. The geostatistical technique we used (ordinary kriging) has the advantage of treating spatial data as the result of a space-indexed family of dependent random variables, strongly linked to their reciprocal spatial location[11]. This is an advantage over other interpolation methods, which assume that data are space independent and handle them as an outcome of random samples or historic series.

The major concern about applying ordinary kriging to such a case proves to be the difficulty of assuming the hypothesis that ozone concentration is stationary in the territory. We use *stationary* here to mean that the expected value of space-indexed variables is the same for all the points in the investigated domain[11,12]. Since ozone concentration in a complex terrain is strongly dependant on transport and local meteorology, the application of this stationary hypothesis proves difficult.

This led us to search for more efficient solutions than the mere application of an ordinary kriging to the examined data. Many different methods were available; however, it seemed interesting to us to verify the applicability of the approach used by Loibl et al.[13], based on a relationship among ozone concentration, altitude, and time of day, within the topographically complex area.

The function these researchers proposed defines the ozone concentrations as depending on time of day and the altitude at the site coordinates (*x, y*):

$$\mathrm{O}(\mathrm{h}_r(x,y),t) = a_1 + a_2\,\mathrm{e}^{-(t-a_3)^2 a_4} \cdot \ln\left(\frac{\mathrm{h}_r(x,y)}{100} + \frac{b_1 t^2 + b_2 t + b_3}{b_4 t^2 + b_5 t + 10000}\,\mathrm{e}^{-b_6 t}\right) \quad (1)$$

where the relative altitude ($h_r[x,y]$) in the position ($x,y$) is the difference between the absolute altitude of ($x,y$) and that of the point located in the lowest position within a range of 5 km. Fig. 2 displays the three-dimensional graph of Eq. 1.

This kind of approach allows relevant simplifications with relation to the complexity of both physical and chemical variables, which influence the spatial and temporal distribution of ozone. However, due to the small size of the study, the influence of local transport and meteorological conditions can be neglected.

The introduction of a function like Eq. 1 allows the isolation of the component linked to altitude when calculating ozone concentration. Following the procedure adopted by Loibl et al.[13], then deducting this component from the measured concentration value, the obtained residual component R($x$,$y$,$t$) is no longer influenced by altitude:

$$\mathrm{R}(x,y,t) = \mathrm{M}(x,y,t) - \mathrm{O}(\mathrm{h}_r(x,y),t) \quad (2)$$

where M($x,y,t$) is the concentration value measured at the ($x,y$) position in $t$ time.

In this case, data are on a weekly basis and therefore the residual portion will be calculated as the weekly mean:

$$\mathrm{R_w}(x,y) = \mathrm{M_w}(x,y) - \mathrm{O_w}(\mathrm{h}_r(x,y)) \quad (3)$$

where $R_w(x,y)$ is the residual value at the site ($x,y$), $M_w(x,y)$ is the weekly concentration value measured at the site ($x,y$), and $O_w(h_r[x,y])$ is the mean weekly value at the site ($x,y$) calculated according to Eq. 1.

Since it is reasonable to assume that the residual component no longer depends on altitude, and since the dependence of ozone concentrations at the ($x,y$) position has been excluded due to the size of the area, the value of the residual portion can be assumed to satisfy the stationary hypothesis.

The 80 × 40 km area for this analysis was selected to match that of a previous study[14] with which we were interested in comparing data, even though we recognised that in some areas of the domain the interpolation would be less reliable than in others. At any rate, the areas that are less densely sampled (and therefore have less reliable results) are the least interesting ones from a forestry point of view, so the overall problem was minimised.

In order to operate the interpolation, the area under study was divided into 2- × 2-km grid cells. According to the model previously explained, the relative altitudes of monitoring sites were defined, as well as those of each cell; then, for each of the selected sites, weekly mean ozone values were calculated depending on ozone relative altitude according to Eq. 1. By subtracting the calculated values from those experimentally measured at each site, the "residual parts" were obtained as defined by Eq. 1, and have then been interpolated by means of an ordinary kriging.

Finally, by summing these interpolated residuals to the values previously obtained by Eq. 1 for each cell of the domain, the weekly concentrations values were estimated on each cell of the investigated area.

The software used for the geostatistical process was Geo-EAS version 1.2.3, developed by US-EPA. The different functions were implemented with FORTRAN routines. The variogram model analysis and the selection of the best fit parameters were made separately on the weekly data for the season.

## AOT40 Maps

Once the estimated values of ozone weekly concentrations were obtained, a procedure for the proper assessment of AOT40 weekly values was then defined. It is worth noting that the AOT40 value, calculated over the spring-summer season, results from summing the AOT40 values calculated on a weekly basis (Eg.4), obtained from the number of hours over 40 ppb as in Eq. 5:

$$\mathrm{AOT40} = \sum_{w=1}^{N} \mathrm{AOT40}_w \quad (4)$$

$$\mathrm{AOT40}_w = \sum_{d=1}^{7} \sum_{\substack{h=1 \\ O_{d,h} > 40}}^{24} \left(O_{d,h} - 40\right) \quad (5)$$

where $O_{d,h}$ is the value of ozone hourly concentration and N is the number of weeks from April to September.

It is quite unlikely that a constant relationship exists between ozone concentrations and AOT40 values calculated on a weekly basis, since the two quantities are from different databases, experimentally obtained under different conditions. As a matter of fact, passive samplers display a concentration pattern integrated in time and varying regularly in the examined period, while the AOT40 index gives the cumulative value of concentrations, beyond a certain threshold, detected during daily hours.

Therefore, data provided by passive samplers can neither differentiate concentrations during the exposure period nor account for diurnal variation, which may strongly affect the AOT40 exposure index.

In conclusion, in order to assess weekly AOT40 values calculated with weekly passive sampler data ($\mathrm{AOT40}_w$), a function which takes into account, hour by hour, the course of ozone concentration must be considered. This is dependent on both time of day and altitude. The value of this function, integrated over a week, must match weekly concentrations actually measured in the considered grid cell.

By applying Eq. 1 to every cell of the domain for each hour of the day, a daily curve of hourly concentration was produced. According to the reasonable assumption related to its invariance in each day of the week, it also represents the mean weekly curve of hourly concentrations.

For each cell and week, this function was therefore calculated so that its average value matched the ozone concentration previously assessed for the same cell and week. Then $\mathrm{AOT40}_w$ values were obtained for each domain cell and for all the considered weeks.

Summing $\mathrm{AOT40}_w$ values for all weeks, a map of expected AOT40 was produced for the whole summer season (from May to August).

This approach may introduce nontrivial errors in the approximations of the index due to the nature of the available data. However, if specific curves C = f (h,t) for the investigated area were available, the introduced error could be dramatically reduced.

The use of hourly ozone concentration data from some monitoring stations supplied with continuous analysers for 1998 were considered but not used to date[15]. These data allowed the calculation of $\mathrm{AOT40}_w$ values for the weeks considered. In the calculation of the AOT40 index on the basis of hourly data, one must keep in mind that the lack of data may affect the final value. Consequently the correction formula indicated in Eq. 5 has been applied:

$$AOT40 = RAOT40 \cdot \frac{N_{tot}}{N_{tot} - N_{miss}} \tag{6}$$

where RAOT40 is the index value calculated with all available data, $N_{tot}$ is the total amount of data for the calculation, and $N_{miss}$ is the amount of missing data.

In this case the formula was applied on a weekly basis instead of a monthly one. The calculated $AOT40_w$ values have been compared with the values assessed for the grid cells containing measurement stations. Results are presented in the "Results and Discussion" section below.

Thanks to the monitoring campaigns performed in previous years[8,15], a series of data sets dating back to 1998 and earlier were available as continuous hourly measurements from analysers and from passive samplers at the same sites. These values have been used to improve the AOT40 assessment. The same procedure mentioned above allowed the comparison of the $AOT40_w$ derived from both passive sampler data and continuous analyser data. $AOT40_w$ values calculated with these two sets of data were related using a regression curve[16]. The regression function has been used to correct $AOT40_w$ values assessed for 1998 in each grid cell. By summing the weekly data thus obtained, a second and more accurate assessment of the index from May to August was generated.

## RESULTS AND DISCUSSION

The passive sampler monitoring in the 15 selected sites (Fig. 1) resulted in the weekly mean concentrations displayed in Table 1. The highest concentrations were recorded in the sites located at highest altitudes, on the south-western border of the investigated area, intercepting the photochemical plume originating from the industrial area surrounding Milan (Fig. 3)[17].

As reported in the previous section, these measurements have allowed the production of maps of weekly concentrations over the whole area. Fig. 4 displays maps related to a 4-week per-

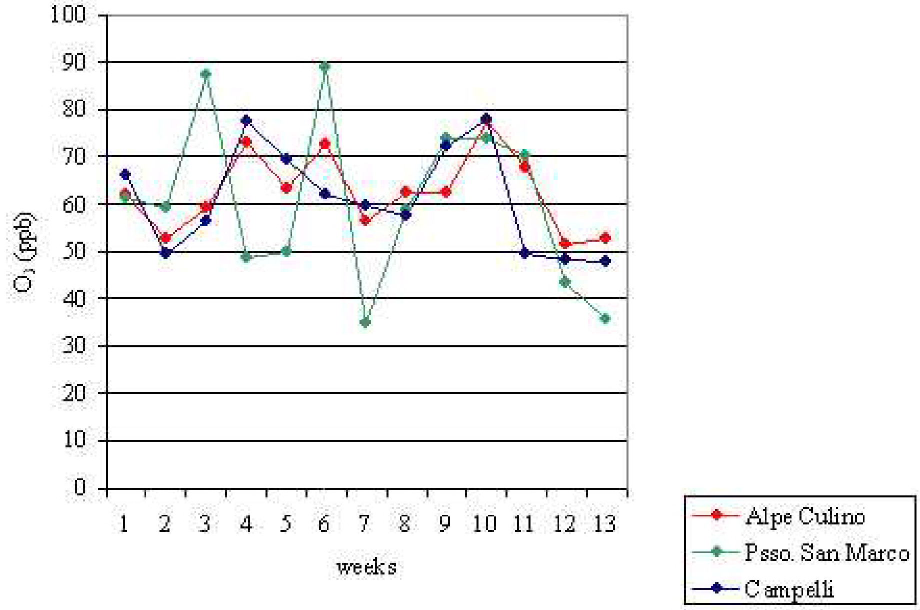

**FIGURE 3.** Ozone weekly concentrations trend in elevated sites (ppb).

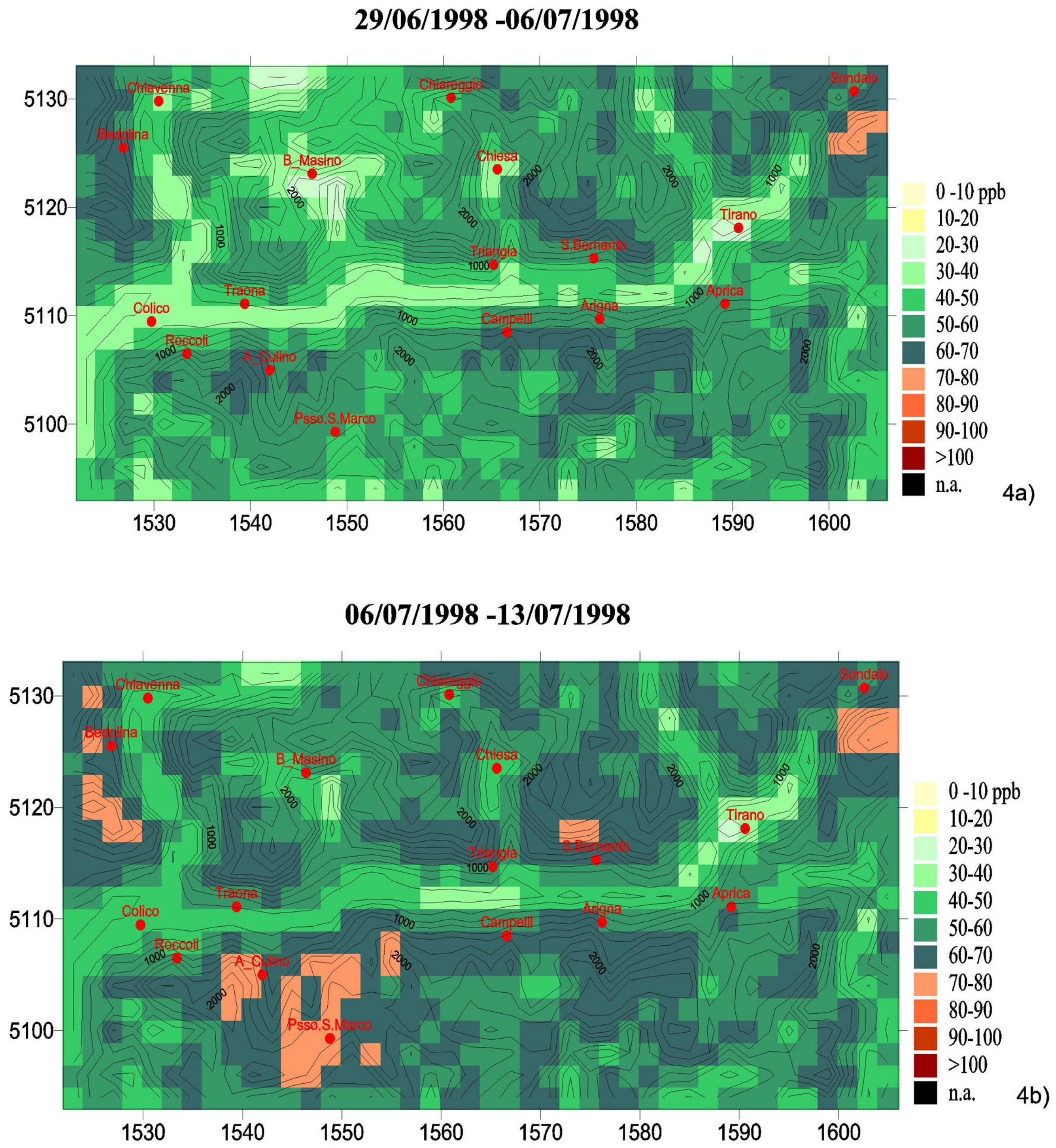

**FIGURE 4.** Ozone weekly concentrations (interpolation procedure described in the text): (a) June 29 to July 6; (b) July 6 to 13; (c) July 13 to 20; (d) July 20 to 27. (Figs. 4c and 4d on following page.)

iod in the month of July. In these maps, the assessed concentration values follow altitude and therefore maintain the orographic outline of the valley, thus confirming the findings discussed in the literature[13,18,19,20,21,22]: ozone concentrations follow a vertical gradient which increases with increasing altitude.

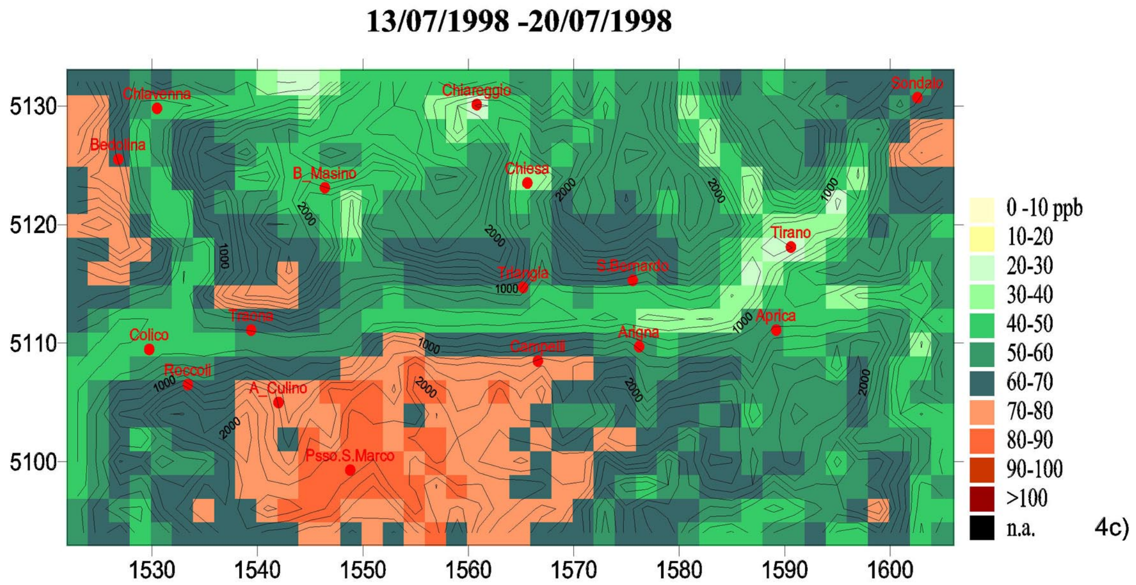

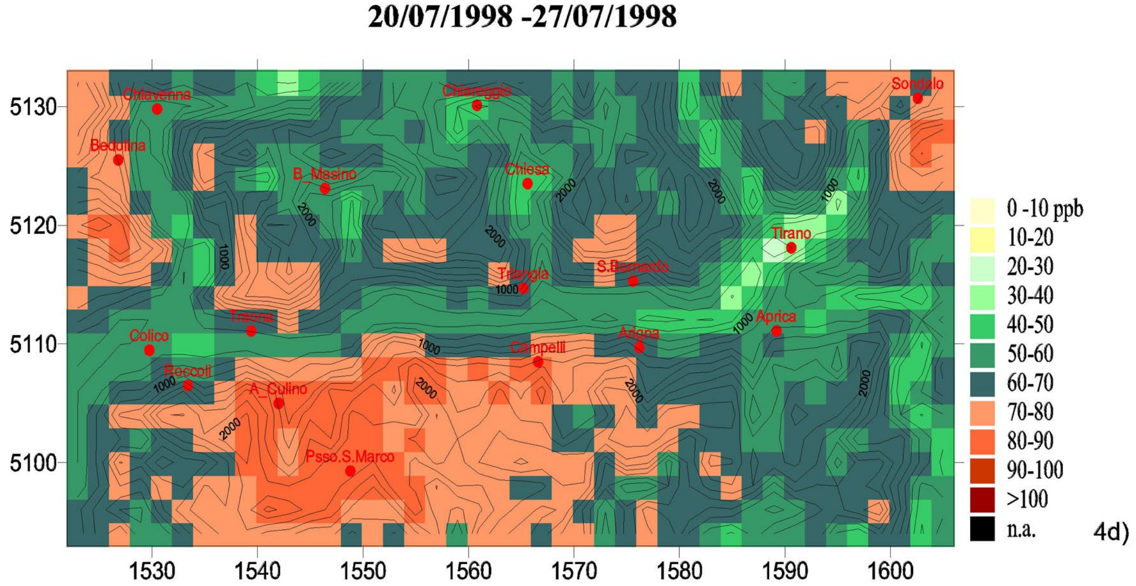

Ozone concentrations assessed in the several weeks show higher values in the south-western portion of the investigated area. Due to the altitude of this area, such elevated concentration values might be ascribed to higher altitude or to mixing of the highest troposphere layers. These mixing phenomena may therefore be of interest only in this elevated area and not affect the whole domain chosen for the interpolation. However, it also likely that in that area ozone (or its precursors) is transported from the underlying major urban and industrial settlements[17]. As a matter of fact, the maps displayed in Fig. 4 seem to indicate an expansion of ozone from sources represented by some border mountain passes between the investigated domain and the industrialised plain. In Pirovano et al.[14], the presence of high ozone concentration in the valley under investigation was ascribed mainly to transport and not to local production, though further analyses are needed to assess the directions of ozone transport in the valley. More sampling locations south of the valley may be required (Fig. 4) to reveal this kind of phenomena.

$AOT40_w$ weekly values were derived for each grid cell according to the procedure described above. The sum of these weekly values produced a map of AOT40 assessment from May to August (Fig. 5). In the graphic, markedly high values of AOT40 are shown; although calculated between May and August, the critical level of 10,000 ppbh established in the Kuopio protocol for the protection of forest vegetation is widely exceeded by up to a factor of 2.5.

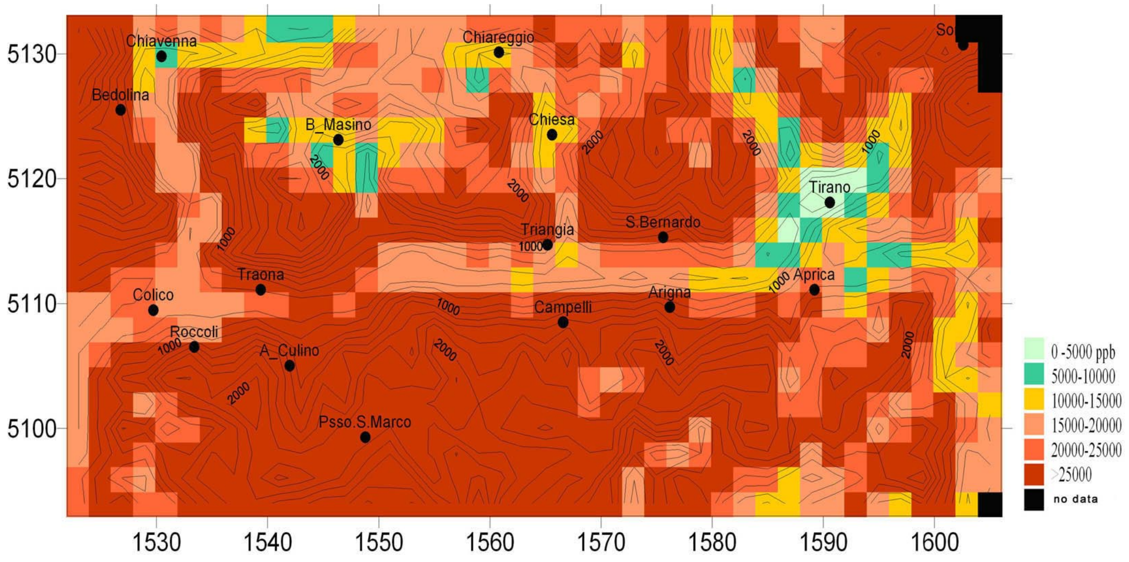

**FIGURE 5.** AOT40 distribution into the domain (uncorrected concentration data).

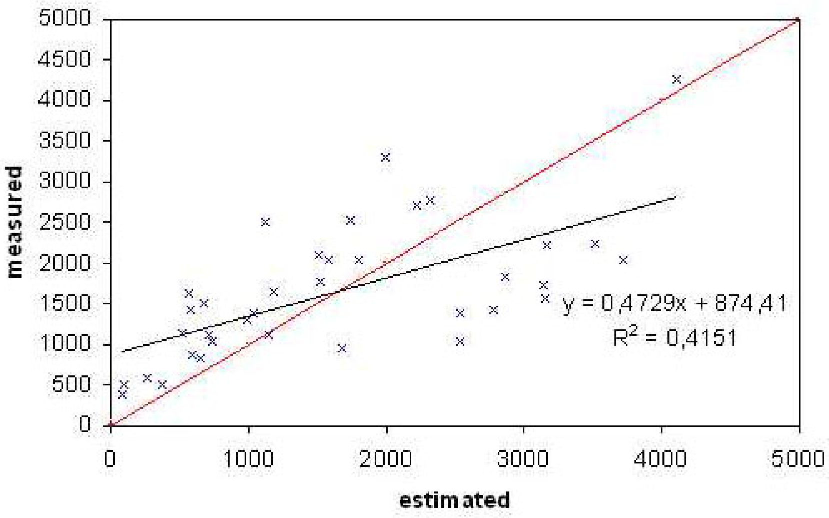

**FIGURE 6.** Measured vs. estimated AOT40 data.

For each grid cell, the assessed AOT40 weekly values have been compared to values calculated with hourly data. This comparison is shown in Fig. 6. The scattering shows that for some weeks the assessed values prove much higher of the actual ones.

By using series of historical data that relate passive and continuous measurements, this preliminary assessment of the AOT40 index values was improved as described in the "Experimental Methods and Computational Procedures" section. The resulting new map is displayed in Fig. 7. The corrected AOT40 values are lower than those previously calculated (Fig. 5), but in any case they are always over the 10,000 ppbh critical level indicated by UN/ECE for forests protection.

The graph in Fig. 8 displays the comparison between this new assessment and the AOT40 values calculated on hourly data previously used (see Fig. 7). The graph shows that new index values, though less scattered than those obtained without corrections (Fig. 6), systematically underestimate the actual values.

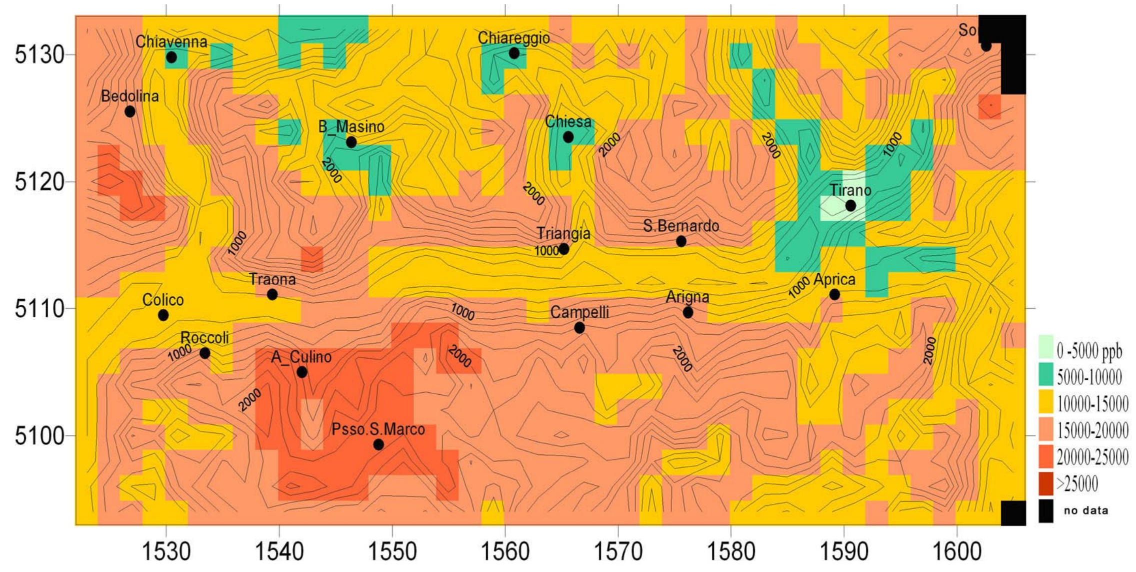

**FIGURE 7.** AOT40 distribution into the domain (corrected concentration data).

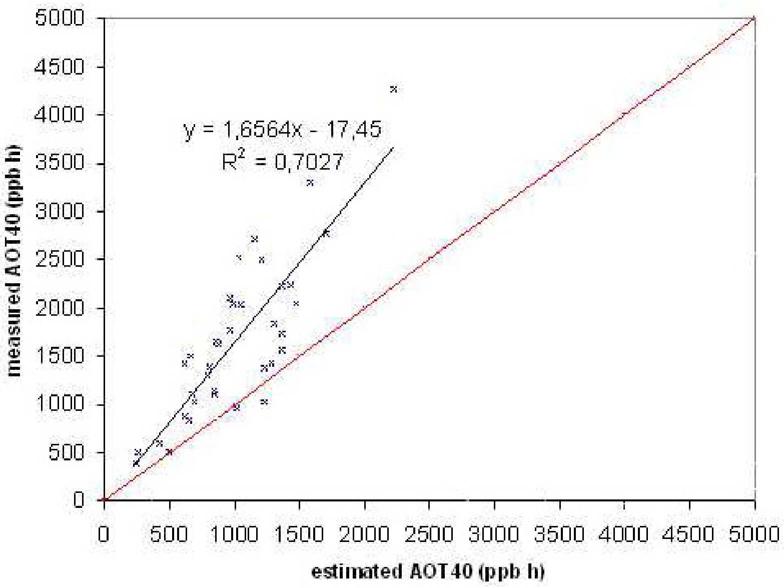

**FIGURE 8.** Measured vs. estimated AOT40 data (corrected as in Fig. 7).

## CONCLUSIONS

Forest damage due to ozone must be considered spatially and not as a series of single-point measurements. Moreover, most investigators adopt potential damage indices (exposure indices) calculated on hourly data over the whole vegetative season. Forests often grow in environments characterized by a complex morphology, where it may be difficult to conduct continuous hourly monitoring over long periods of time and in enough sites to allow extrapolation over the whole terrain studied.

The present study aimed to develop a methodology for the assessment of potential damage indices on the whole investigated terrain on the basis of integrated measurements and the use of passive samplers. Therefore, the present investigation can be regarded as a pilot study aimed at testing the efficiency of passive sampling of air pollutants for ozone risk assessment based on exposure indices as an alterative to the use of automatic analysers.

The results show that this quantitative approach using passive samplers allows risk assessments that may be useful to air pollution control policy makers in agriculture and forest ecosystems management.

The results obtained in this preliminary study could be improved by using a function similar to Eq. 1 calculated specifically for the investigated area. This function has not been calculated mainly because of the lack of hourly ozone profiles in enough sites located at different altitudes. The use of a nonspecific curve for the investigated area has been partly compensated by the correction of AOT40 assessments on the basis of available historic data series, though these may also be partly affected by the poor variation in site altitudes.

The use of an exposure index for which a critical level has been associated with injury allowed the highlighting of potential damage to forest vegetation in the investigated area. In fact, the final AOT40 values estimated prove to be lower in some sites than those calculated by hourly measurements. Although the assessment covers a period from May to August and not the April to September period suggested by the Kuopio protocol, the 10,000 ppbh critical level is exceeded in most areas.

Therefore, regional policies aiming at the protection of most vegetation species sensitive to ozone and its related photochemical pollutants may be grounded on a rational and scientific basis.

## ACKNOWLEDGEMENTS

This project has been successfully carried out thanks to the collaboration of several institutions (Fondazione Lombardia per l'Ambiente, A.R.P.A. Città di Milano, A.R.P.A. and Provincia di Sondrio, Ecosfera S.r.l. and Linnæa S.n.c.) and has been financially supported by Fondazione Lombardia per l'Ambiente and Provincia di Sondrio.
